# A case report of challenges in distinguishing gastroesophageal junction hepatoid adenocarcinoma from testicular germ cell tumor: Insights for improved diagnosis with gene expression profiling

**DOI:** 10.1177/2050313X231223469

**Published:** 2024-01-03

**Authors:** Omid Yazdanpanah, Fa-Chyi Lee, Roozbeh Houshyar, Mahra Nourbakhsh, Nataliya Mar

**Affiliations:** 1 Division of Hematology and Oncology, UC Irvine Medical Center, Orange, CA, USA; 2 Department of Radiology, UC Irvine Medical Center, Orange, CA, USA; 3 Department of Pathology, UC Irvine Medical Center, Orange, CA, USA

**Keywords:** Gastric cancer, hepatoid adenocarcinoma, germ cell tumor, gene expression profiling

## Abstract

Gastroesophageal junction hepatoid adenocarcinoma is a rare form of gastroesophageal cancer. We present a case of a 38-year-old man with no significant medical history who was diagnosed with gastroesophageal junction hepatoid adenocarcinoma but initially misdiagnosed with a testicular germ cell tumor, given the elevated alpha-feto protein and poorly differentiated pathology. We will elaborate on the importance of gene expression profiling in modern oncology to better define the tumor of origin in patients with cancer of unknown primary origin, how it helped us to diagnose gastroesophageal junction hepatoid adenocarcinoma and how it can help identify potential additional therapeutic targets in some cases. Due to the rarity of this subtype of gastroesophageal junction cancer there is a lack of standard therapeutic options, and we will discuss the most commonly used treatment regimens. The patient underwent three lines of antineoplastic therapy and unfortunately passed after 51 weeks of follow-up.

## Introduction

Gastroesophageal junction hepatoid adenocarcinoma (GEJHA) is an uncommon subtype of gastroesophageal cancer.^1^ It has similar histopathological features to hepatocellular carcinoma (HCC) and is associated with an elevated alfa-fetoprotein (AFP).^2^ AFP is a nonspecific marker that can be elevated in malignancies such as tumors of gonadal origin (both germ cell and non-germ cell tumors), HCC, or gastric cancer^3–5^ as well as chronic liver diseases, including viral hepatitis.^6^

In cases where tumors are poorly differentiated and an accurate pathologic diagnosis to pinpoint the tumor of origin is difficult, gene expression profiling assays may be extremely valuable.^7^ By utilizing reverse transcriptase polymerase chain reaction (RT-PCR) or gene microarray techniques, these approaches can determine the primary tumor location and help in finding actionable mutations for prognostic and therapeutic purposes.^7^

We report a case of a patient, who presented with multiple liver and lung metastases, but no clear primary site of malignancy, which was initially thought to be an extragonadal germ cell tumor and was ultimately discovered to be GEJHA based on molecular tumor analysis.

## Case presentation

**Figure 1.**
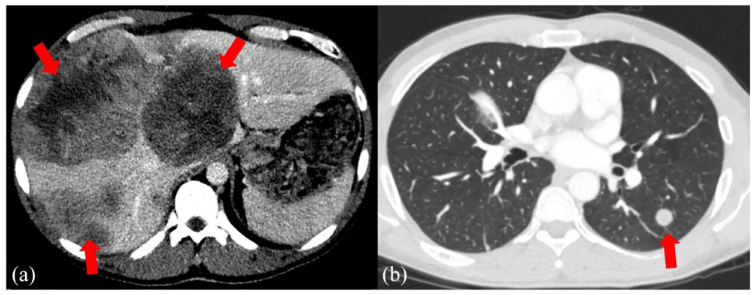
(a) Computed tomography (CT) scan of the abdomen and pelvis: Liver enlargement and multiple heterogeneous hypodense masses depicted with arrows (largest 11 cm). (b) CT scan of the chest: Multifocal left lower lobe pulmonary nodules depicted with arrow (largest 1.6 × 1.4 cm) and pulmonary emboli.

A 38-year-old man with no significant past medical history presented to the emergency department with worsening right upper quadrant abdominal pain, low-grade fevers, and dyspnea accompanied by 10 kg weight loss in the past 3 months prior to presentation (Time 0). He had a 20-pack-year smoking history and heavily used alcohol for 13 years until 2 years prior to the current presentation. Computed tomography (CT) of the abdomen and pelvis with contrast revealed hepatomegaly with multiple heterogeneous hypodense masses, measuring up to 11 cm and concerning for metastasis (Figure 1(a)). Native chest CT showed multiple pulmonary nodules, with the largest measuring 1.6 cm, and pulmonary emboli (Figure 1(b)).

**Figure 2.**
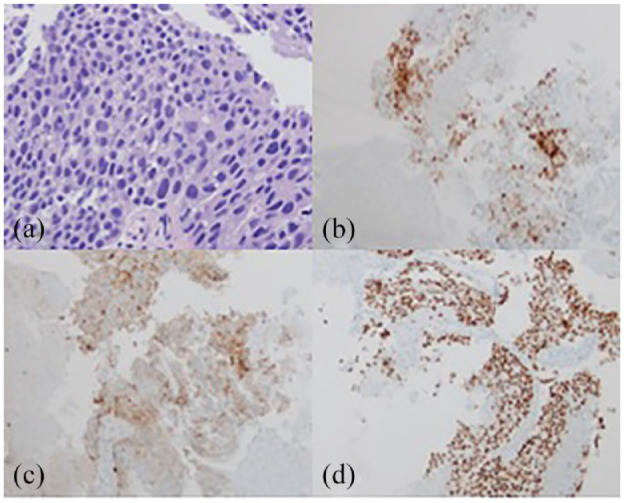
Liver mass biopsy. (a) H&E staining indicative of poorly differentiated carcinoma. (b) Positive CDX2 staining. (c) Positive Glypican 3 staining. (d) Positive Sal-like protein 4 staining.

Labs were remarkable for AFP of 61,395 ng/ml (reference range: <9.0 ng/mL) and beta human chorionic gonadotropin of 8.1 (reference range: <2 mIU/mL). Serologic workup for hepatitis A, B, C, and HIV was negative. He underwent a hepatic mass biopsy a week after initial presentation (Time +1w), with pathology demonstrating poorly differentiated malignancy with predominantly solid growth pattern and patchy necrosis. Immunohistochemistry (IHC) was positive for Sal-like protein 4 (SALL4), Glypican3 (GPC3), cytokeratin AE1/AE3 (AE1/3), caudal-type homeobox transcription factor 2 (CDX2), and alpha-fetoprotein (AFP) (Figure 2) favoring yolk sac tumor.^8^ The following markers displayed negative staining: Oct4, Arginase1, HepPar1, Chromogranin, Napsin A, NKX3.1, TTF-1, CK5/6, EMA, P63, SOX10, and PAX8.

**Figure 3.**
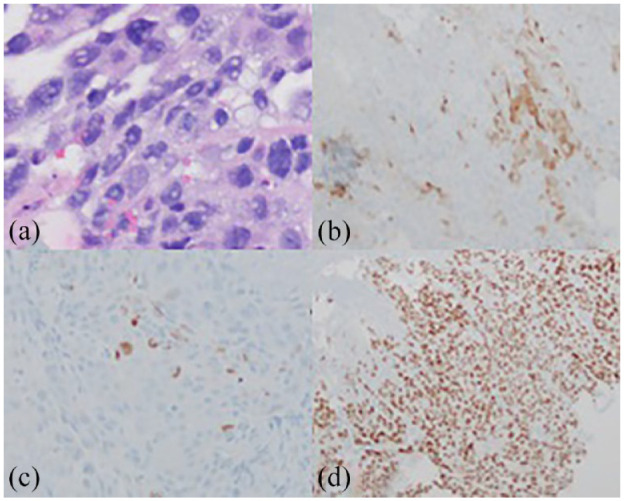
Gastroesophageal junction mass biopsy. (a) H&E staining indicative of poorly differentiated carcinoma. (b) Positive Arginase staining. (c) Rare positivity for Hepar1 staining. (d) Positive Sal-like protein 4 staining.

After case discussion at a multidisciplinary tumor board, the patient was initiated on chemotherapy for presumed germ cell tumor with etoposide, ifosfamide, and cisplatin (VIP regimen with 21 days cycles) (Time +2w). After two cycles of chemotherapy, he had clinically improved with controlled decreased abdominal pain as well as resolution of anorexia, dyspnea, and low-grade fevers. His AFP decreased to 18,463 ng/ml (Time +8w). At this time, a previously ordered Tempus Tumor Origin assay resulted as 84% probability of gastroesophageal carcinoma. Meanwhile, Tempus xT tissue next generation sequencing (NGS) assay revealed a microsatellite stable tumor with a tumor mutational burden of 3.2, and Programmed Death-Ligand 1 Combined Positive Score of <1. NGS also revealed a *TP53 p.R248W* missense variant mutation, *ERBB3 (HER3)* overexpression, and *CDKN2A* copy number loss. The patient then underwent an esophagogastroduodenoscopy (EGD) (Time +9w), which demonstrated an ulcerated mass at the gastroesophageal junction (GEJ). Pathology showed poorly differentiated carcinoma, which stained positive for cytokeratin AE1/3, GPC3, CDX2, and SALL4 as well as patchy positivity for arginHepar1, with a differential diagnosis of HCC with GEJ metastasis versus GEJHA (Figure 3). Given the clinical response to VIP chemotherapy, the patient was continued on the same regimen and completed four cycles of treatment. His AFP subsequently reached a plateau in the 18,000 ng/ml range, while restaging CT scans showed a partial treatment response (Time +14w).

**Figure 4.**
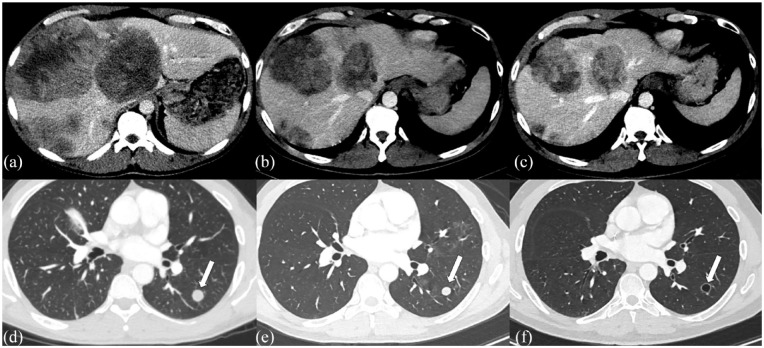
CT scan of chest, abdomen, and pelvis. Hepatic and pulmonary metastatic lesions response to treatment. (a) Multiple liver masses at the time of diagnosis. (b) Liver lesions following four cycles of etoposide + ifosfamide + cisplatin (VIP regimen). (c) Liver lesions following two cycles of FLOT (5-FU, leucovorin, oxaliplatin, docetaxel) plus pembrolizumab. (d) Multiple left lower lobe pulmonary nodules (largest indicated with arrow) at the time of diagnosis. (e) Lung nodules following four cycles of VIP regimen. (f) Lung nodules following two cycles of FLOT plus pembrolizumab.

The patient’s chemotherapy regimen was then changed to 5-fluorouracil (5-FU), leucovorin, oxaliplatin, and docetaxel (FLOT regimen) with pembrolizumab for presumed GEJHA^9^ (Time +16w). After three cycles of FLOT chemotherapy, his AFP decreased to 7,867 ng/ml. Restaging CT scans showed further decrease in hepatic and lung lesions and no new sites of disease (Figure 4) (Time +22w). Patient received three additional cycles of the FLOT regimen, but subsequent CT imaging showed disease progression in the liver (Time +28). Patient was also hospitalized with Staphylococcus aureus bacteremia. Following recovery from infection, he underwent embolization of a large right hepatic dome lesion and was then was initiated on dual checkpoint blockade with ipilimumab and nivolumab (Time +33w). After 3 months of this treatment, his liver metastasis had worsened on CT and AFP rose to 49,827 ng/ml (Time +45w). Clinically, his previous cancer-related symptoms worsened and he required recurrent hospitalizations for sepsis, poor nutrition, and liver failure. As his performance status had declined significantly, the patient was transitioned to hospice and passed away (Time 51w).

## Discussion

Cancer of unknown primary site (CUP) comprises about 3%–5% of all invasive cancer diagnoses in the United States.^10^ All efforts should be aimed toward identifying the most specific diagnosis to select the most effective therapy. Routine light microscopy and histology alone cannot determine the origin of the tumor or distinguish between treatment sensitive and resistant cancers.^11^ However, IHC may be very helpful in determining the tumor lineage such as carcinoma versus lymphoma versus sarcoma by using antibodies directed at specific cell components or products.^12,13^ In nearly 95% of CUPs, standard IHC examination can identify the tumor’s origin, although a small subset of these cases will be categorized as poorly differentiated or undifferentiated neoplasms.^14^ These cases can be challenging and IHC can occasionally even be misleading, such as in our patient where IHC was initially suggestive of a yolk sac tumor. This can result in anchoring bias, defined as a tendency to fixate on the evidence found very early in the diagnostic process while ignoring additional information and leading to premature conclusions.^15–17^

Gene expression profiling and molecular cancer classifier assays are valuable novel diagnostic tools in the evaluation of CUP when standard pathologic evaluation and IHC are equivocal. These modalities can predict the original tumor site by analyzing tumor-specific gene expression signatures using RT-PCR or gene microarray techniques.^7^ These assays can successfully identify the tumor’s origin in up to 95% of carcinoma cases.^18^ In our patient, the tumor of origin testing was suggestive of a GEJ adenocarcinoma, which led to additional workup with an EGD that ultimately identified the correct diagnosis. In hindsight, GEJHA is prone to liver metastasis and is also a high secretor of AFP.^1^ Gene expression profiling can also be useful in finding actionable genetic alterations for prognostic and therapeutic purposes in some CUP tumors. In our patient, overexpression of ERBB3 (HER3) was identified and may have been a potential therapeutic target, although the patient did not survive long enough to receive this class of agents.

GEJHA is considered to be an aggressive malignancy, with a worse prognosis than gastric adenocarcinoma.^19^ It is a rare malignancy, comprising only 1%–2% of gastric cancers.^20^ Although the optimal therapeutic approach for this malignancy remains controversial,^21^ this is generally a platinum-sensitive malignancy, which may explain our patient’s initial response to VIP chemotherapy that is commonly used for the treatment of germ cell tumors. A systematic review based on 18 cases reported that first-line cisplatin-based chemotherapy in patients with GEJHA yielded a 75% response rate.^22^ Other active agents for this malignancy include combinations of 5-fluorouracil, irinotecan, docetaxel, paclitaxel, methotrexate, and mitomycin C.^21,23–25^ There are also rare case reports supporting the use of immune checkpoint inhibitors in GEJHA.^26^ As such, our patient received FLOT chemotherapy with pembrolizumab in the second-line setting, with a transient treatment response.

*ERBB2 (HER2)* overexpression is observed in about 25% of patients with GEJ adenocarcinoma and addition of trastuzumab to chemotherapy is associated with improved overall survival.^27^ However, our patient had *ERBB3 (HER3)* overexpression, which is noted in up to 16% in gastric adenocarcinomas.^28^ *ERBB3 (HER3)* overexpression is associated with higher grade tumors and more aggressive clinical behavior as well as resistance to oncolytics that target other HER receptors such as EGFR (epidermal growth factor receptor) and HER2 inhibitors.^23,24^ Tucatinib is a tyrosine kinase inhibitor that targets both ERBB2 (HER2) and ERBB3 (HER3), which has shown efficacy in treatment of HER2-positive unresectable or metastatic breast^29,30^ and colorectal cancer.^31^ However, it is unclear if it is beneficial in HER3 overexpressed tumors in the absence of HER2 alteration. There is also no data with this agent in GEJHA. In our case, patient’s declining performance status did not allow introduction of another line of therapy. However, obtaining NGS to identify additional therapeutic targets is important in this tumor type given the lack of standard therapeutic options.

## Conclusion

Gene expression profiling is an essential tool in modern oncology to better characterize the tumor of origin in patients with CUP. Although not always feasible, making the strongest attempt to identify the source of malignancy is crucial to select optimal therapies. GEJHA is a rare and aggressive subtype of GEJ cancers, with a lack of standard therapeutic options. Obtaining NGS in patients with GEJHA is essential to help identify potential additional therapeutic targets.
